# Laryngeal mask airway Unique™ position in paediatric patients undergoing magnetic resonance imaging (MRI): prospective observational study

**DOI:** 10.1186/s12871-018-0617-2

**Published:** 2018-10-24

**Authors:** Jozef Klučka, Jan Šenkyřík, Jarmila Skotáková, Roman Štoudek, Michaela Ťoukalková, Ivo Křikava, Lukáš Mareček, Tomáš Pavlík, Alena Štouračová, Petr Štourač

**Affiliations:** 1 Department of Paediatric Anaesthesia and Intensive care, University Hospital Brno, Faculty of medicine, Brno, Czech Republic; 2 Department of Paediatric Radiology, University Hospital Brno, Faculty of Medicine, Masaryk University, Brno, Czech Republic; 3 Faculty of Medicine, Masaryk University Brno, Brno, Czech Republic; 4 Institute of Biostatistics and Analyses, Faculty of Medicine, Masaryk University Brno, Brno, Czech Republic; 5 Department of Radiology and Nuclear Medicine, Faculty of Medicine, Masaryk University and University Hospital Brno, Brno, Czech Republic

**Keywords:** Laryngeal mask, Position, Magnetic resonance imaging, Paediatric anaesthesia

## Abstract

**Background:**

Laryngeal mask UNIQUE® (LMAU) is supraglottic airway device with good clinical performance and low failure rate. Little is known about the ideal position of the LMAU on the magnetic resonance imaging (MRI) and whether radiological malposition can be associated with clinical performance (audible leak) in children. The primary aim of the study was to evaluate incidence of the radiologic malposition of the LMAU according to size. The secondary outcome was the clinical performance and associated complications (1st attempt success rate, audible leak) in LMAUs in correct position vs. radiologically misplaced LMAUs.

**Methods:**

In prospective observational study, all paediatric patients undergoing MRI of the brain under general anaesthesia with the LMAU were included (1.9.2016–16.5.2017). The radiologically correct position: LMAU in hypopharynx, proximal cuff opposite to the C1 or C2 and distance A (proximal cuff end and aditus laryngis) ≤ distance B (distal cuff end and aditus laryngis). Malposition A: LMAU outside the hypopharynx. Malposition B: proximal cuff outside C1-C2. Malposition C: distance A ≥ distance B. We measured distances on the MRI image. Malposition incidence between LMAU sizes and first attempt success rate in trainees and consultant groups was compared using Fisher exact test, difference in incidence of malpositions using McNemar test and difference in leakage according to radiological position using two-sample binomial test.

**Results:**

Overall 202 paediatric patients were included. The incidence of radiologically defined malposition was 26.2% (*n* = 53). Laryngeal mask was successfully inserted on the 1st attempt in 91.1% (*n* = 184) cases. Audible leak was detected in 3.5% (*n* = 7) patients. The radiologically defined malposition was present in 42.9% (*n* = 3) cases with audible leak. The rate of associated complications was 1.5% (n = 3): laryngospasm, desaturation, cough. In 4.0% (*n* = 8) the LMAU was soiled from blood.

Higher incidence of radiological malposition was in LMAU 1.0, 1.5 and LMAU 3, 4 compared to LMAU 2 or LMAU 2.5 (*p* < 0.001).

**Conclusion:**

Malposition was not associated with impaired clinical performance (audible leak, complications) of the LMAU or the need for alternative airway management.

**Trial registration:**

Clinicaltrials.gov  (NCT02940652) Registered 18 October 18 2016.

## Background

Laryngeal mask (LMA) has gained significant popularity in the past decades also in paediatric anaesthesia. Nowadays, LMA is being used in whole spectrum of surgical procedures, including ENT and laparoscopic surgery [1]. Laryngeal mask has several advantages compared to endotracheal tube due to it’s supraglottic position – it can reduce the laryngospasm, cough and the incidence of postoperative desaturation [2]. The failure rate is minimal, the learning curve is steep and fast and the clinical performance appears to be almost perfect [3, 4]. The Laryngeal mask airway Unique® is widely used disposable laryngeal mask, easy to insert and in paediatric patients performs similar to or even better than the laryngeal mask airway Classic [5–7]. Despite all the advantages, the ideal anatomical position on the magnetic resonance imaging (MRI) remains to be controversial due to lack of data. Goudsouzian et al. [8] defined correct position of the LMA based on the results of the observational study (50 children undergoing computer tomography or MRI) as the proximal cuff lies opposite to the C1 or C2 vertebrae however the position of the distal cuff of the LMA, contrary to the Brain recommendation (correct distal position opposite to the C6 or C7 vertebrae) [9], was located between C4 and T1 vertebrae. Another evaluation of the radiologically correct position was published by Monclus et al. [10], where the authors describe the position of the Ambu AuraOnce mask in 121 children who underwent MRI. The correct position was defined as the distance from the proximal cuff end to the laryngeal entrance (aditus laryngis) (distance A) was smaller or equal to the distance from the distal cuff end to the laryngeal entrance (distance B). The MRI examination in majority of paediatric patients is being performed under general anaesthesia due to limited cooperation of the paediatric patients (based on the age of the patients) and the need of movement suppression during the whole MRI scanning. Airway in paediatric patients undergoing MRI exam is predominantly secured by LMA, due to lower invasivity and lower rate of associated complications compared to tracheal tube [11].

The primary aim of the study was to evaluate incidence of the radiologic malposition of the LMA according to size of the LMA. The secondary outcome was the clinical performance of the LMA and associated complications (1st attempt success rate, audible leak) in LMAs in correct position vs. radiologically misplaced LMAs.

## Methods

**Fig. 1:**
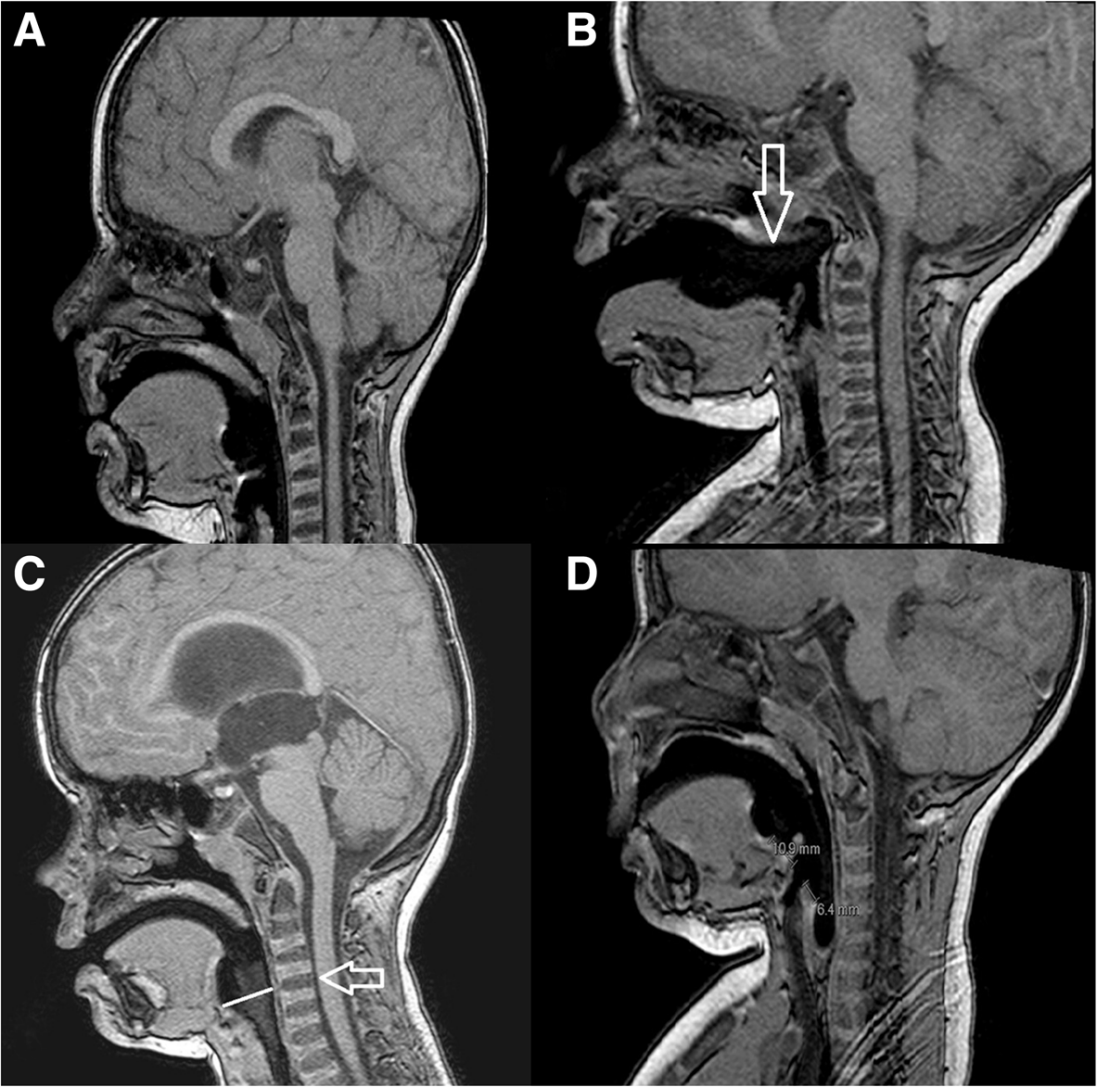
Laryngeal masks positions on MRI image. **a** Correct position of the LM; **b** Malposition A; **c** Malposition B; **d** Malposition C

Prospective observational study was approved by the local ethics committee (Ethics committee by University Hospital Brno, approved 9/2016). The consent for the anonymous use of collected data for scientific purposes was obtained from the participants. We registered study on ClinicalTrials.gov (NCT02940652). Paediatric patients (age between 28 days – 19 years) in selected period (1. 9. 2016–16. 5. 2017) undergoing MRI of the brain or brain and cervical spine under general anaesthesia with the airway secured by laryngeal mask were included in the study. Patients outside the age limits, extreme weight categories (under 1 kg, over 120 kg), patients in risk of malignant hyperthermia, with the need of vasopressor or inotropic therapy due to circulatory instability and patients with high aspiration risk (hiatal hernia, ileus, gastroesophageal reflux) were excluded. Inhalation induction was preferred in small children (under 8 years old). In children with intravenous line in situ, the bolus dose of propofol was used to suppress the excitation stage during inhalation induction. Because of the observational character of the study, general anaesthesia was induced and maintained according to the physician (pragmatic study). We used laryngeal mask based on the recommendation from manufacturer (size according to the weight of the patients, volume of the inflated air in the seal ring) and we didn’t measure the intracuff pressure. The LMA mask type (Unique®, Classic®) could be selected for airway management, based on the physician´s preference. After anaesthesia induction, with the head of the patient in the neutral position, according to the clinician decision (in case of intravenous induction after diminish of eylid/blink reflex or/and after apnoe onset, in case of inhalation induction after diminish of eylid/blink reflex and after intravenous line was placed and secured), the LMAU was inserted with classical technique, partially inflated, with the lubricated tip of the mask (clear liquid-based lubricating jelly). Based on the previously published data, the radiologically correct position was defined as LMA location in hypopharynx, proximal cuff of the LMA opposite to the C1 or C2 vertebrae and the distance between proximal cuff end and aditus laryngis (distance A) ≤ distance from distal cuff end and aditus laryngis (distance B) (Fig. 1a). Malposition of the LMA outside the hypopharynx was defined as Malposition A (Fig. 1b). Malposition of the proximal cuff outside C1-C2 was defined as Malposition B (Fig. 1c). Malposition C was defined as the distance A ≥ distance B (Fig. 1d). The distances were measured on the MRI image (Panorama HFO by Philips (high field open MRI scanner) field strength 1.0 T, T1 sagittal sequence). The primary outcome was the incidence of the radiological malposition according to the size of the mask. The secondary outcome was the clinical performance of the LMA (1st attempt success rate, audible leak, peak leak pressure – measured only in patients on mechanical ventilation) and to compare the clinical performance of the LMA classified as radiologically misplaced with clinical performance of the LMAs located in the radiologically correct position. According to the previously published trials the leak was measured by presence of audible air leak. The peak pressure was measured only in mechanically ventilated patients by increasing the inspiratory pressure until the audible leak was detected. The statistical significance of differences in laryngeal mask malposition between LMA sizes was assessed using the Fisher exact test. The modelling of simultaneous effect of LMA size on laryngeal mask malposition was carried out by univariate logistic regression model (OR was calculated compared to reference group LMA 2). The Standard level of statistical significance α = 0.05 was used. The first attempt success rate in trainees and consultant groups was compared using Fisher exact test. Significant difference in the incidence of laryngeal mask malpositions A, B and C was evaluated using McNemar test. Two-sample binomial test was used for evaluation of difference in the laryngeal mask leakage according to its radiological position. All computations were performed by using R software version 3.4.0 [12].

## Results

In the selected study period, 220 patients were included and after exclusion of 18 patients (8.2%, insufficient data, MRI of other body regions, age outside defined limit) 202 were eligible for the final analysis. The median age of the study cohort was 3 years (30 days – 16 years) and the median weight was 15 kg (2.5 – 60 kg). Anaesthesia was maintained with sevoflurane (median inspiratory concentration 1.8%) with nitrous oxide or without nitrous oxide in 73.3% vs. 26.7% (148 vs. 54). All laryngeal masks (100%, 202/202, LMA Unique®) were properly inserted on the first attempt in 91.1% (*n* = 184) with the lowest success in LMAU size 1 subgroup 71.4% (5/7) followed by LMAU size 2.5–84% (42/50). The first attempt success rate was higher in trainees compared to consultants (98.1% vs. 88.5%, *p* = 0.047). In 88.6% (*n* = 179) cases the patients were spontaneously breathing and the remaining 11.4% (*n* = 23) were mechanically ventilated (pressure-controlled ventilation – 6.4%, *n* = 13; pressure-support ventilation – 4.0%, *n* = 8; volume-controlled ventilation 1.0%, *n* = 2). The radiological malposition outside hypopharynx – malposition A (on MRI view) was detected in 2% (*n* = 4) patients. The malposition B (proximal cuff not opposite to the C1 or C2 vertebrae) was identified in 5.4% (*n* = 11) cases and the malposition C (distance A > distance B) was detected in 19.8% (*n* = 40) cases. The total frequency of LMAU in radiologically incorrect position (malposition A + B + C) were 26.2% (*n* = 53, there were 2 combined malposition A + B and A + C in two patients). The incidence of malposition C was statistically significant more frequent compared to A and/or B (both *p* < 0.0001), however there were no significant difference between incidence of malposition A and B (*p* = 0.096). The lower incidence of the malposition was in LMAU size 2 and 2.5 (18% and 28%) (Table 1).

**Table 1:** Incidence of the radiologically defined malposition vs. correct position according to the size of LMAU

LMAU size	Malposition (A + B + C)		Radiologically correct position		*p*-value
	N	%	n	%	
LMAU 1.0	1	14.3%	6	85.7%	< 0.001
LMAU 1.5	10	50.0%	10	50.0%	
LMAU 2.0	20	18.0%	91	82.0%	
LMAU 2.5	14	28.0%	36	72.0%	
LMAU 3.0	3	37.5%	5	62.5%	
LMAU 4.0	4	100.0%	0	0.0%	

Explanatory text: 2 patients with unspecified LMAU size were excluded from analysis (one LMAU was in malposition A and one in radiologically correct position)

The calculated OR for malposition risk was higher in smaller LMAU (1 and 1.5, OR: 3.1, 95% CI: 1.3–7.8) and bigger LMAU size (3 and 4, OR: 6.4, 95% CI: 1.8–22.1) when compared to LMAU 2 as the reference group (Table 2).

**Table 2:** Laryngeal masks UNIQUE® with leak according to the radiological position

LMAU size	Malposition (A + B + C)		Radiologically correct position		*p*-value
	N	%	n	%	
LMAU 1.0 + 1.5	11	40.7%	16	59.3%	0.004
LMAU 2.0	20	18.0%	91	82.0%	
LMAU 2.5	14	28.0%	36	72.0%	
LMAU 3.0 + 4.0	7	58.3%	5	41.7%	
	OR		95% IS		*p*-value
LMAU 2.0	1.00		–		–
LMAU 1.0 + 1.5	3.13		1.26–7.75		0.014
LMAU 2.5	1.77		0.81–3.88		0.15
LMAU 3.0 + 4.0	6.37		1.83–22.12		0.004

Explanatory text: 2 patients with unspecified LMAU size were excluded from analysis

The rate of overall radiological malposition (A + B + C) was higher in physicians in training, compared to consultants (37.0% vs. 22.2%, *n* = 20/54 vs. *n* = 33/148, *p* = 0.046). The audible leak was detected in 3.5% (*n* = 7) cases (5 cases with spontaneous breathing and 2 cases with pressure control ventilation – PCV). Radiological malposition was detected in 42.9% (n = 3) of the cases with the presence of audible leak (1x LMAU size 1 – malposition C. 2x LMAU size 2 – malposition A and/or malposition C) see Table 3.

**Table 3:** Laryngeal masks UNIQUE® with leak according to the radiological position

	Malposition overall (A + B + C) (*n* = 53)	Radiologically correct position (*n* = 149)	*p* value
Leak	5.6% (*n* = 3)	2.7% (*n* = 4)	*p* = 0.309

There were no statistically significant difference in the presence of audible leak in the subgroup of LMAUs in radiologically defined malposition (A + B + C, n = 3/53, 5.6%) and in the LMA in radiologically correct position (*n* = 4/149, 2.7%, *p* = 0.309).

The peak seal pressure was measured in 10.9% (*n* = 22) patients (patients on mechanical ventilation). The median peak pressure was 24 cmH_2_0 (18–48 cmH_2_0). After removal, the LMAU was soiled with mucus in 9.9% (*n* = 20) and blood in 4.0% (*n* = 8). Associated complications were detected in 1.5% cases (n = 3) – laryngospasm, cough and desaturation. We reported no single case of gastric contents regurgitation and the need for alternative airway management due to failure of the LMAU.

## Discussion

Most important finding of our study is also concordant with results of Goudsouzian et al. [8] and Monclus et al. [10], where the radiologically defined malposition of the LMA did not have impact on clinical performance of the laryngeal mask too. Our results support the definition of the correct position of the LMA defined by Goudsouzian [5] and Vialet [11] - proximal cuff opposite to the cervical vertebrae C1 or C2. In view of our results, the position of the distal cuff cannot be taken into account, when considering the ideal position of the LMAU due to the high variability (from C3 to T2 vertebrae, Goudsouzian et al. [5] – C4-T1). In all patients in the cohort the LMAU was successfully introduced and the overall 1st attempt success rate (91.1%) of LMAU introduction is comparable to previously published data by Lopez-Gil et al. [3, 4] (90%) and Pournajafian et al. [13] (80.6%), however higher success rate was reported when using Ambu AuraOnce mask (95% first attempt success) [10]. The higher incidence and higher OR for overall malposition in smaller LMAU (size 1 and 1.5) is consistent with Monclus et al. [10], however also higher incidence and OR in bigger LMAU (size 3 and 4) was detected in our cohort. LMAU size 3 + 4, however represents only 3.4% (*n* = 7) patients from the whole cohort, so there can be high risk of bias. One of the possible explanations of the higher detected 1st attempt success rate in trainees compared to consultants can be the daily anaesthesiology routine of trainees, compared to standard daily consultant practice. The possible explanation for the higher incidence of malposition in trainees could be the lower incidence of reposition and higher satisfaction rate with the 1st attempt insertion. LMAU is considered safe supraglottic airway with minimal failure rate, easy to use with the steep learning curve [3, 4]. Performance of the laryngeal mask remains to be almost ideal, which is underlined by the 0% failure rate in our study cohort, with no need for alternative airway management. Laryngeal mask has currently firm and predominant position in the supraglottic airway devices group; it carries many advantages when compared to the endotracheal tube - lower incidence of cough, desaturation and laryngospasm [2], however still, after 34 years from Dr. Brain’s pilot study [14], the ideal position is not well defined. Based on previous data [7] we confirmed that LMAU can be in radiologically misplaced in relatively high percentage of patients (26.2%), however the clinical impact of radiological malposition remained consistently nonsignifficant.

The clinical performance of the LMAU is based on presence of audible leak during the spontaneous or mechanical ventilation and access the peak seal pressure of the system. In the study cohort the measured mean seal pressure of the system (24 cm H_2_0) and the minimal audible leak incidence (3.5%) is consistent with reported results [8, 10, 11] and supports the estimated high efficacy and low failure rate of LMAU in paediatric anaesthesia, which in combination with the minimal rate of associated complications (1.5%) in the study cohort further highlight the position of the LMAU in the airway management in paediatric anaesthesia patients. Results of the study can lead to further investigation, whether the radiological malposition can have impact on the seal of the gastric contents and therefore influence the safety of anaesthesia with LMAU for airway management.

### Limitations

The main limitation is observational character of the study and the definition of the correct Laryngeal mask position based on previously spare data and data based on different Laryngeal mask type (Ambu AuraOnce®) compared to LMA UNIQUE® used in the study. Another limitation is the low rate of mechanical ventilation subgroup in our cohort and therefore low number of patients with seal pressure measured, however the study was designed as observational study and the primary aim was to compare the radiologically defined LMAU malposition according to the size of LMA and to compare the clinical performance of the LMAU in the standard clinical conditions.

## Conclusion

Radiologically defined malposition on MRI view was more frequent in smaller LMAU (1, 1.5) and bigger LMAU (3, 4) compared to LMAU size 2 and 2.5 and did not have impact on clinical performance of the LMA Unique® in paediatric patients undergoing MRI in general anaesthesia with airway secured by the laryngeal mask.

## References

[1] Goldmann K (2006). Recent developments in airway management of the paediatric patient. Curr Opin Anaesthesiol.

[2] Luce V, Harkouk H, Brasher C (2014). Supraglottic airway devices vs tracheal intubation in children: a quantitative metaanalysis of respiratory complications. Paediatr Anaesth.

[3] Lopez-Gil M, Brimacombe J, Alvarez M (1996). Safety and efficacy of the laryngeal mask airway. A prospective survey of 1400 children. Anaesthesia.

[4] Lopez-Gil M, Brimacombe J, Cebrian J, Arranz J (1996). Laryngeal mask airway in pediatric practice: a prospective study of skill acquisition by anesthesia residents. Anesthesiology.

[5] Jagannathan Narasimhan, Ramsey Melissa A., White Michelle C., Sohn Lisa (2015). An update on newer pediatric supraglottic airways with recommendations for clinical use. Pediatric Anesthesia.

[6] Verghese C, Berlet J, Kapila A, Pollard R (1998). Clinical assessment of the single use laryngeal mask airway—the LMA-unique. British Journal of Anaesthesia.

[7] Brimacombe J, von Goedecke A, Keller C, Brimacombe L, Brimacombe M. The laryngeal mask airway unique versus the soft seal laryngeal mask: a randomized, crossover study in paralyzed, anesthetized patients. Anesth Analg. 2004;99(5):1560–3; table of contents. 10.1213/01.ANE.0000133916.10017.6D 15502065

[8] Goudsouzian Nishan G., Denman William, Cleveland Robert, Shorten George (1992). Radiologic Localization of the Laryngeal Mask Airway in Children. Anesthesiology.

[9] Brain A.I.J. (1992). Laryngeal mask misplacement?causes, consequences and solutions. Anaesthesia.

[10] MONCLUS ENRIC, GARCÉS ANTONIO, DE JOSE MARIA BELEN, ARTÉS DAVID, MABROCK MAGED (2007). Study of the adjustment of the Ambu laryngeal mask under magnetic resonance imaging. Pediatric Anesthesia.

[11] VIALET RENAUD, NAU ANDRÉ, CHAUMOÎTRE KATHIA, MARTIN CLAUDE (2008). Effects of head posture on the oral, pharyngeal and laryngeal axis alignment in infants and young children by magnetic resonance imaging. Pediatric Anesthesia.

[12] R Core Team. R: a language and environment for statistical computing. Vienna: R Foundation for Statistical Computing; 2017. URL http://www.R-project.org/.

[13] Pournajafian A, Alimian M, Rokhtabnak F, Ghodraty M, Mojri M. Success rate of airway devices insertion: laryngeal mask airway versus supraglottic gel device. Anesth Pain Med. 2015;5(2):e22068. 10.5812/aapm.22068. 10.5812/aapm.22068 PMC4389102 25866709

[14] BRAIN A.I.J. (1983). THE LARYNGEAL MASK—A NEW CONCEPT IN AIRWAY MANAGEMENT. British Journal of Anaesthesia.

